# Fe-based hybrid electrocatalysts for nonaqueous lithium-oxygen batteries

**DOI:** 10.1038/s41598-017-09982-9

**Published:** 2017-08-25

**Authors:** Seun Lee, Gwang-Hee Lee, Hack Jun Lee, Mushtaq Ahmad Dar, Dong-Wan Kim

**Affiliations:** 1 School of Civil, Environmental and Architectural Engineering, Korea University, Seoul, 02841 Republic of Korea; 2 Center of Excellence for Research in Engineering Materials, Advanced Manufacturing Institute, College of Engineering, King Saud University, Riyadh, 11421 Saudi Arabia

## Abstract

Lithium–oxygen batteries promise high energy densities, but are confronted with challenges, such as high overpotentials and sudden death during discharge–charge cycling, because the oxygen electrode is covered with the insulating discharge product, Li_2_O_2_. Here, we synthesized low–cost Fe–based nanocomposites via an electrical wire pulse process, as a hybrid electrocatalyst for the oxygen electrode of Li–O_2_ batteries. Fe_3_O_4_-Fe nanohybrids–containing electrodes exhibited a high discharge capacity (13,890 mA h g_c_^−1^ at a current density of 500 mA g_c_^−1^), long cycle stability (100 cycles at a current rate of 500 mA g_c_^−1^ and fixed capacity regime of 1,000 mA h g_c_^−1^), and low overpotential (1.39 V at 40 cycles). This superior performance resulted from the good electrical conductivity of the Fe metal nanoparticles during discharge–charge cycling, which could enhance the oxygen reduction reaction and oxygen evolution reaction activities. We have demonstrated the increased electrical conductivity of the Fe_3_O_4_-Fe nanohybrids using electrochemical impedance spectroscopy.

## Introduction

Transition metal oxides (TMOs) are effective catalysts in various fields, such as water splitting^1^, fuel cells^2^, wastewater treatment^3^, biomedical applications, and energy storage^4–6^ as electrocatalysts, TMOs enable rechargeable batteries with high energy densities, for use in the energy storage systems of smart grids and electric vehicles. TMO electrocatalysts are one of the key technologies in the area of lithium–oxygen (Li–O_2_) batteries^7–10^. Li–O_2_ batteries are confronted by several critical problems, including a high overpotential during discharge–charge cycling, low rate capability, and capacity fading owing to the sluggish kinetics of the oxygen reduction reaction (ORR) and oxygen evolution reaction (OER) during discharge and charge cycling^11–13^. In particular, during the discharge process, the oxygen electrode is covered with Li_2_O_2_, which is intrinsically electronically insulating and does not provide the electrodes with sufficient electronic conductivity, resulting in sudden death^14, 15^. As a result, Li–O_2_ batteries have capacity limitations. Therefore, electrocatalysts should be developed to significantly improve reversibility.

Among the TMO candidates, Fe_3_O_4_ is considered a promising electrocatalyst owing to its high abundance, low cost, and excellent catalytic performance, which is better than that of other TMOs^16–19^. Fe_3_O_4_ has good electronic conductivity and excellent catalytic activity for oxygen reduction due to continuous electron exchange between the octahedrally coordinated Fe(II) and Fe(III) positions via electron hopping^20^. However, although numerous recent studies on TMO electrocatalysts have exhibited improved cycle performance, the effect of the electrochemical performance derived from Fe_3_O_4_ on the oxygen electrodes for Li–O_2_ batteries has been rarely reported^21–23^.

Previous reports suggest that metal or carbon based iron oxide nanocomposites prepared the highly active electrocatalyst in the Li–O_2_ batteries^23–25^. Fe_3_O_4_/Fe core-shell electrocatalysts for Li–O_2_ batteries were uniformly dispersed on the surface of the carbon support, with the porous structure of carbon well persevered and high catalytic activity towards the ORR and OER^23^. However, it is still a challenge to design electrocatalysts that can drastically improve the efficiency and cycling ability of current Li–O_2_ batteries.

Herein, we report a facile method for obtaining Fe_3_O_4_-Fe nanohybrids via an electrical wire pulse process. The Fe_3_O_4_-Fe nanohybrids showed excellent catalytic activity for both the ORR and OER during discharge–charge cycling. The obtained Fe_3_O_4_-Fe nanohybrids exhibited enhanced catalytic activity relative to Fe_3_O_4_ nanospheres and manifested superior electrode performance, including a high specific capacity (13,890 mA h g_c_^−1^ at 500 mA g_c_^−1^), low overpotential (1.39 V at 500 mA g_c_^−1^), and excellent high rate stability (150 cycles at 2,000 mA g_c_^−1^ with a fixed capacity regime of 1,000 mA h g_c_^−1^).

## Results and Discussion

**Figure 1:**
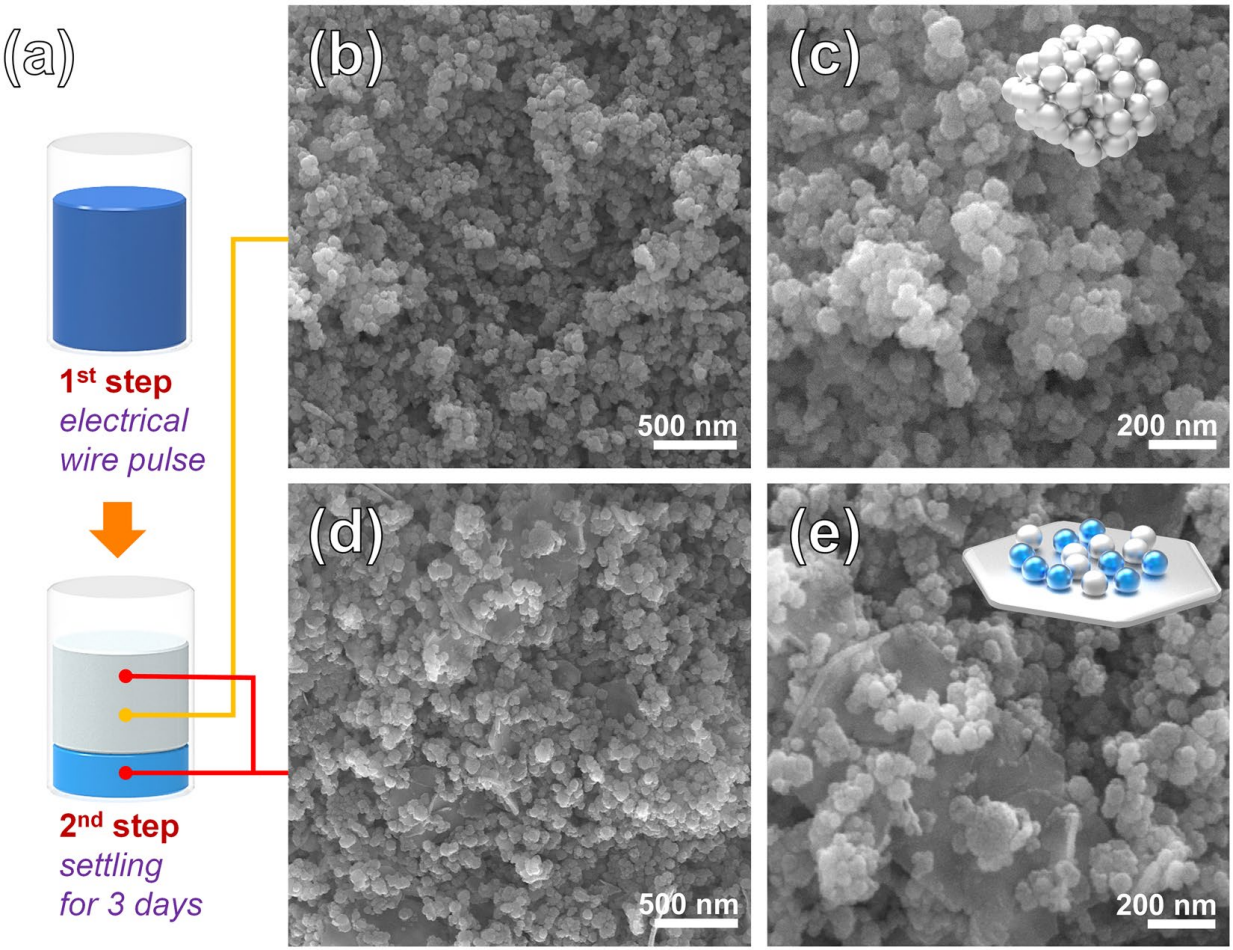
Morphology analysis of the Fe_3_O_4_ nanospheres and the Fe_3_O_4_-Fe nanohybrids. (**a**) Scheme illustration of selection process of the Fe_3_O_4_ nanospheres and the Fe_3_O_4_-Fe nanohybrids. FESEM images of (**b,c**) the Fe_3_O_4_ nanospheres and (**d,e**) the Fe_3_O_4_-Fe nanohybrids.

Figure 1 shows a schematic of the selection process and typical field emission scanning electron microscopy (FESEM) images of the products obtained using the electrical wire pulse method. After the selection step, in which the suspension was allowed to settle, two different morphologies were obtained (Fig. 1b–e). After settling for 3 days, Fig. 1b and c show typical FESEM images of the Fe_3_O_4_ nanospheres obtained from the upper-colloid solution, which have with very homogeneous shapes. Figure 1d and e show typical FESEM images of the Fe_3_O_4_-Fe nanohybrids obtained from the whole colloid solution, which have nanospheres as well as thin nanoflakes.

**Figure 2:**
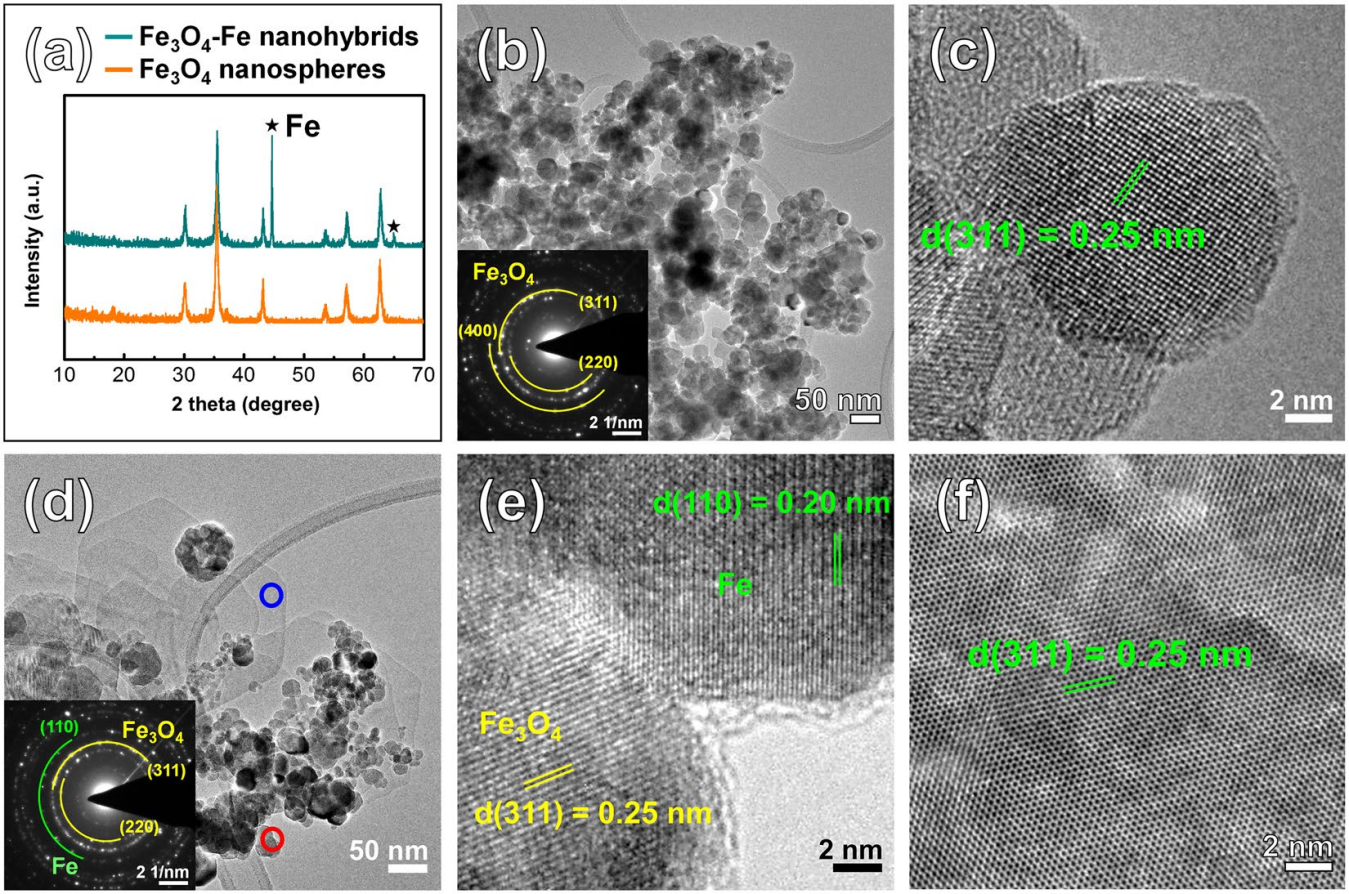
Structure and component analysis of the Fe_3_O_4_ nanospheres and the Fe_3_O_4_-Fe nanohybrids. (**a**) XRD patterns of Fe_3_O_4_ nanospheres and the Fe_3_O_4_-Fe nanohybrids. (**b**) Low-magnitude TEM images and SAED pattern (inset) and (**c**) HRTEM image of the Fe_3_O_4_ nanospheres. (**d**) Low-magnitude TEM images and SAED pattern (inset) of the Fe_3_O_4_-Fe nanohybrids, and (**e**) HRTEM image of the nanosphere indicated by red circle of (**d**), (**f**) HRTEM image of the nanoflake indicated by blue circle of (**d**).

The crystal structures of the Fe_3_O_4_ nanospheres and the Fe_3_O_4_-Fe nanohybrids were recorded using XRD, as shown in Fig. 2a. The X-ray diffraction (XRD) pattern of the Fe_3_O_4_ nanospheres can be indexed to cubic Fe_3_O_4_ (PDF card No. 88-0315). No signals from other impurities could be clearly detected, indicating that a high purity magnetite sample was prepared from the upper-colloid solution after settling. The XRD pattern of the Fe_3_O_4_-Fe nanohybrids had diffraction peaks that were similar to those of the Fe_3_O_4_ nanospheres. In addition, new diffraction peaks at 44.7° and 65° were observed, which could be attributed to cubic Fe (PDF card No. 06-0696).

To further confirm the morphology and structure of the Fe_3_O_4_ nanospheres and the Fe_3_O_4_-Fe nanohybrids, TEM images were obtained, as shown in Fig. 2b–f. Figure 2b and c show typical transmission electron microscopy (TEM) images of the Fe_3_O_4_ nanospheres, with a similar morphology to that observed by SEM. The Fe_3_O_4_ nanospheres had diameters of less than 50 nm and were aggregated with an approximately spherical shape. The selected area electron diffraction (SAED) pattern demonstrates the highly crystalline nature of the nanoparticles (inset of Fig. 2b). The lattice spacing, calculated based on the electron diffraction patterns, are in agreement with that of cubic Fe_3_O_4_ (PDF card No. 88-0315). The high-resolution TEM (HRTEM) image in Fig. 2c shows that the inter-planar distance in the Fe_3_O_4_ nanospheres is 0.25 nm, which is consistent with the (311) plane of cubic Fe_3_O_4_.

The TEM images of the Fe_3_O_4_-Fe nanohybrids reveal 0D spheres deposited on 2D flake composites (Fig. 2d). The SAED pattern of the nanocomposites can be indexed to cubic Fe_3_O_4_ (PDF card No. 88-0315) and cubic Fe (PDF card No. 06-0696), which is consistent with the XRD results. Figure 2e shows the HRTEM image of a selected nanosphere in Fig. 2d (indicated by the red circle), and reveals the presence of two different types of nanospheres. The inter-planar distance of 0.25 nm corresponds to the (311) plane of Fe_3_O_4_, whereas the other region with an inter-planar distance of 0.20 nm is indexed to the (110) plane of Fe. The energy-dispersive X-ray spectroscopy (EDS) mapping profile of the Fe_3_O_4_-Fe nanohybrids, as shown in Supplementary Fig. S1, indicating that Fe and O elements are respectively populated in the Fe_3_O_4_-Fe nanohybrids. Supplementary Fig. S1b show the EDS element color mixing mapping of the Fe_3_O_4_-Fe nanohybrids. The element mapping images in Supplementary Fig. S1b–d reveal the presence of O (purple) and Fe (yellow) elements, as determined from the EDS analysis. The Fe nanospheres are evenly distributed in the Fe_3_O_4_-Fe nanohybrids (Supplementary Table S1). Figure 2f shows the HRTEM image of a selected nanoflake in Fig. 2d (indicated by the blue circle), and reveals the highly crystalline nature of the nanoflakes. The inter-planar distance of the nanoflakes is 0.25 nm, consistent with the (311) plane of cubic Fe_3_O_4_. The folded edge or protuberant ridge of the Fe_3_O_4_ nanoflakes (see Supplementary Fig. S2a) demonstrate an average thickness of approximately 10 nm, suggesting that the nanoflakes comprise octahedral Fe_3_O_4_ arranged with hexagonal symmetry in the planar direction. The Fe_3_O_4_ flakes are laterally grown with cracked surfaces, as shown in Supplementary Fig. S2b. As this result, the surface of the Fe_3_O_4_ nanoflakes can be expected to cause defects. So we measured surface chemistry using O 1 s XPS analysis (Supplementary Fig. S3). Comparing the Fe_3_O_4_-Fe nanohybrids and the Fe_3_O_4_ nanoflakes of O 1 s XPS spectra, the O 1 s peak of the Fe_3_O_4_-Fe nanohybrids is lower binding energy than that of the Fe_3_O_4_ nanospheres. Shift of the O 1 s peak to lower binding energy indicates oxygen vacancy^26^. Oxygen vacancy generated the charge of the defect state, which can expected vigorous electrocatalytic activity.

The Fe_3_O_4_ nanoflakes can be achieved by oriented attachment growth. The oriented attachment growth is contributed to reduce the overall energy of the formed Fe_3_O_4_ nanocrystals. In other words, when the growth of Fe_3_O_4_ seeds through a kinetically controlled process ceases, the 2D hexagonal flakes grows into more thermodynamically favored shape according to different surface facets of different surface energy^27, 28^. Fe_3_O_4_ has a cubic inverse spinel structure which consists of a face-centered-cubic (FCC) close-packed structure with oxygen anions. As a FCC close-packed structure, the surface energies corresponding to different surface facets usually increase in the order of γ_{111}_ < γ_{100}_ < γ_{110}_. In this crystal structure, the Fe_3_O_4_ crystals usually exist with {111} planes as the basal surfaces.

In the electrical wire pulse method, when a high rate of energy is injected by a pulse with a high-density current, the fine metal wire is heated in a liquid solvent, and the generated energy leads to evaporation and condensation of the metal as a highly dispersed nanocolloids. The nanocolloids quickly grow through vapor cooling in the solvent^29, 30^. In case of our work, the oriented attachment growth would occurs to form hexagonal Fe_3_O_4_ nanoflakes at early growth stage by rapid hot-injection provided from a pulse with a high-density current. At high temperature, high energy facets will lead to a fast growth rate compared to low energy facets. During rapid pulsed explosion process, the Fe_3_O_4_ nanospheres and the Fe_3_O_4_-Fe nanohybrids are produced because of repeated energy injection. As can be seen Supplementary Fig. S4, typical TEM images of the Fe_3_O_4_-Fe nanohybrids are observed the attachments between aggregated particles, even larger ones. A hexagonal shaped aggregate can be seen being formed by the aggregation step (Supplementary Fig. S4a). This aggregated particles will continue to undergo oriented attachment growth to form a polycrystalline structure and subsequent recrystallization to a single crystal since the low surface energy of the {111} facet can no longer compensate for excessive strain energy (Supplementary Fig. S4b). Actually, both the oriented attachment growth and Ostwald ripening occur simultaneously^31, 32^. In the early growth stages of hot-injection, the strong surface adsorption lead to the oriented attachment growth, and the Ostwald ripening is thermodynamically disturbed. Consequently, in later growth stage, large Fe_3_O_4_ nanospheres and Fe_3_O_4_ nanoflakes are formed by the Ostwald ripening.

**Figure 3:**
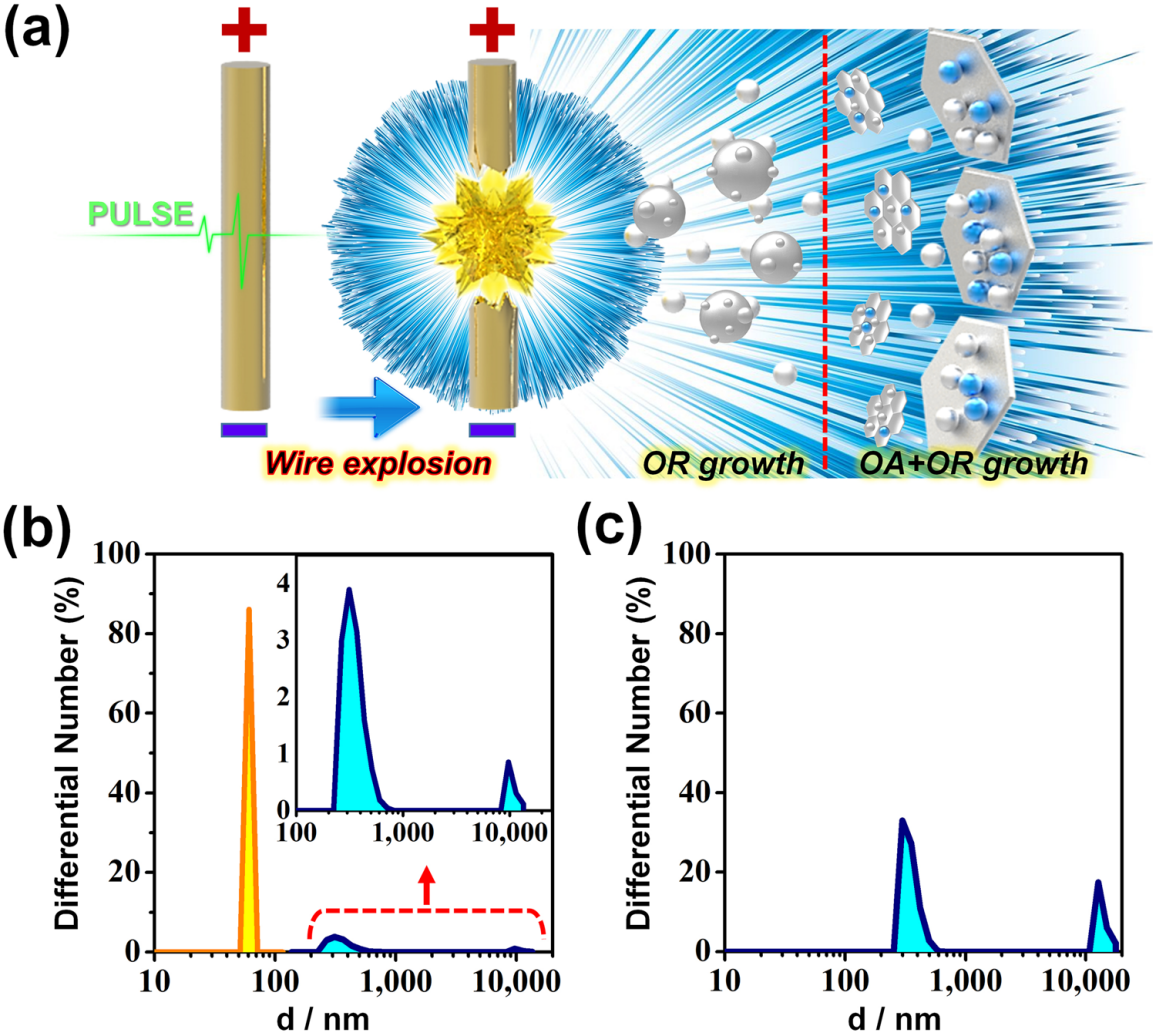
(**a**) Schematic illustration of growth mechanism of the Fe_3_O_4_-Fe nanohybrids (oriented attachment (OA) + Oswald ripening (OR) growth) and Fe_3_O_4_ nanospheres (OR growth) by the electrical wire pulse process. Particle size distribution of (**b**) the Fe_3_O_4_-Fe nanohybrids and (**c**) the Fe_3_O_4_ nanospheres.

The particle size distribution of the Fe_3_O_4_-Fe nanohybrids and Fe_3_O_4_ nanospheres can be obtained by dynamic light scattering analysis after adequate ultrasonic treatment. We observed three peaks for the Fe_3_O_4_-Fe nanohybrids around 60 nm, 310 nm, and 9 μm (Fig. 3b). However, the hydrodynamic diameters of the Fe_3_O_4_ nanospheres were concentrated at 300 nm and 12 μm, with no peak around 60 nm (Fig. 3c). Because of the good dispersibility of the Fe_3_O_4_ nanoflakes, it is assumed that the hydrodynamic diameters of the Fe_3_O_4_-Fe nanohybrids are concentrated at 60 nm. After settling for 3 days, the Fe_3_O_4_ nanospheres were obtained from the upper-colloid solution. In contrast, the Fe_3_O_4_-Fe nanohybrids, which are both Fe_3_O_4_ and Fe nanospheres deposited on the Fe_3_O_4_ nanoflakes, sank to the lower-colloid solution owing to the different densities of Fe (7.9 g cm^−3^) and Fe_3_O_4_ (5.2 g cm^−3^) (see Supplementary Table S2).

**Figure 4:**
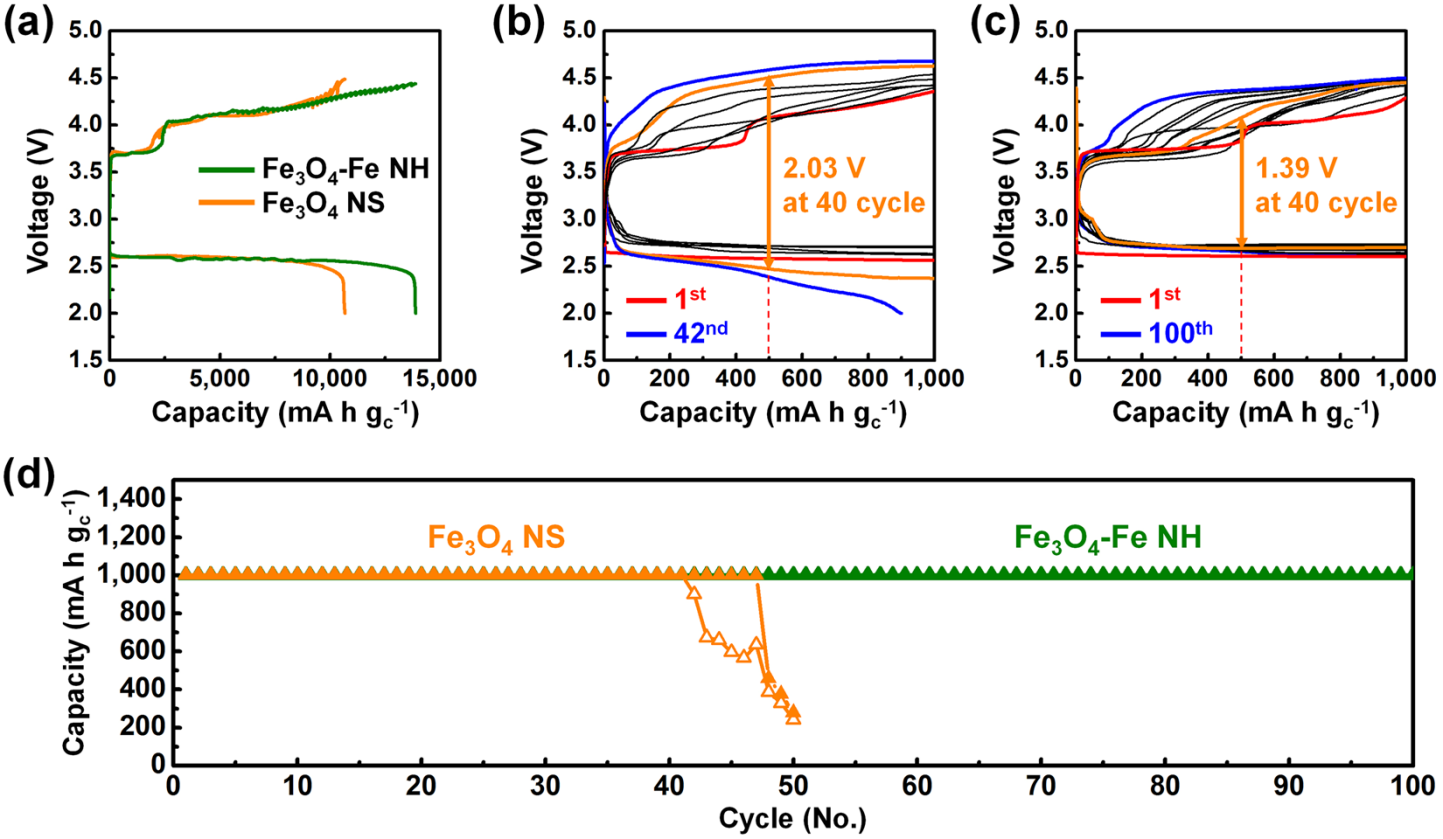
Electrochemical performances of the Fe_3_O_4_-Fe NH and the Fe_3_O_4_ NS electrode at a current rate of 500 mA g_c_^−1^. (**a**) Galvanostatic discharge-charge curves for the Fe_3_O_4_-Fe NH electrode and the Fe_3_O_4_ NS electrode. (**b**) The Fe_3_O_4_ NS electrode and (**c**) the Fe_3_O_4_-Fe NH electrode at a fixed capacity regime of 1,000 mA h g_c_^−1^ with a rate of 500 mA g_c_^−1^, (**d**) Comparison of the discharge-charge specific capacity versus the cycle number of the Fe_3_O_4_-Fe NH electrode and the Fe_3_O_4_ NS electrodes.

In this work, we tested Li–O_2_ batteries with an oxygen electrode as the cathode, which consisted of Super P carbon black (SP) and the Fe_3_O_4_-Fe nanohybrids or Fe_3_O_4_ nanospheres as electrocatalysts (weight ratio of 1:1). The first galvanostatic discharge–charge cycle of the Fe_3_O_4_-Fe nanohybrid-containing electrode (Fe_3_O_4_-Fe NH electrode) and the Fe_3_O_4_ nanosphere-containing electrode (Fe_3_O_4_ NS electrode) are shown in Fig. 4a at a current rate of 500 mA g_c_^−1^ (equivalent to 500 mA g_electrocatalyst_^−1^). The most significant result was observed on cycling in the 2.0–4.8 V window. The Fe_3_O_4_-Fe NH electrode exhibited a discharge capacity of approximately 13,890 mA h g_c_^−1^, which was superior to that of the Fe_3_O_4_ NS electrode (10,680 mA h g_c_^−1^). The morphological changes after discharge and charge at a current rate of 500 mA g_c_^−1^ for the Fe_3_O_4_-Fe NH and Fe_3_O_4_ NS electrodes are shown in Supplementary Fig. S5. After discharge process, ORR aggregates (Li_2_O_2_) were covered fully on surface of the Fe_3_O_4_-Fe NH electrode (Supplementary Fig. S5a). The ORR aggregates disappeared at the end of the charge process (Supplementary Fig. S5b), indicating the high reversibility of the Fe_3_O_4_-Fe NH electrode. However, the Fe_3_O_4_ NS electrode after discharge process are formed toroidal shaped ORR aggregates and were covered incompletely (Supplementary Fig. S5c). At the end of the charge process, the ORR aggregates were not fully decomposed (Supplementary Fig. S5d). The Fe_3_O_4_ NS electrode indicates to operate the irreversible discharge-charge process.

The cyclic voltammograms (CV) for the Fe_3_O_4_-Fe NH and Fe_3_O_4_ NS electrodes are shown in Supplementary Fig S6. Both the anodic and cathodic peaks for the Fe_3_O_4_-Fe NH electrode were significantly larger than those for the Fe_3_O_4_ NS electrode over five cycles. These results demonstrate the promising ORR/OER activity of the Fe_3_O_4_-Fe NH electrode as an electrocatalyst in the Li–O_2_ battery. The peak above 4.15 V in the first charge process likely corresponds to the decomposition of the carbon-containing electrolyte. The peak of the Fe_3_O_4_ NS electrode is obviously higher than that of the Fe_3_O_4_-Fe NH electrode, which should be related to the poor cycle reversibility^33, 34^.

Figure 4b–d show the galvanostatic discharge–charge cycle performance of Li–O_2_ cells with the Fe_3_O_4_-Fe NH and Fe_3_O_4_ NS electrodes at a current rate of 500 mA g_c_^−1^ and at a fixed capacity regime of 1,000 mA h g_c_^−1^. As can be seen Fig. 4c, the first voltage plateau of the Fe_3_O_4_-Fe NH electrode is around 3.75 V. The second voltage plateau jumps suddenly to around 4.00 V but remains relatively stable, reaching 4.29 V at the end of the charging process. Ganpathy *et al*. observed two Li_2_O_2_ oxidation stage during charge process^35^. At low voltage plateau, the amorphous Li_2_O_2_ is decomposed, whereas at high voltage plateau, crystalline Li_2_O_2_ is decomposed starting with the smaller crystals that evolves oxygen via a Li deficient solid-solution reaction. The overpotential of the Fe_3_O_4_-Fe NH electrode is lower than that of the Fe_3_O_4_ NS electrode during discharge–charge cycling. The galvanostatic discharge–charge curve of the Fe_3_O_4_-Fe NH electrode at 40 cycles (Fig. 4c) revealed a remarkably low overpotential (1.39 V) compared with that of the Fe_3_O_4_ NS electrode (2.03 V) (Fig. 4b). Indeed, the Fe_3_O_4_-Fe NH electrode could effectively enhance the electrocatalyst kinetics over 100 cycles (Fig. 4d).

**Figure 5:**
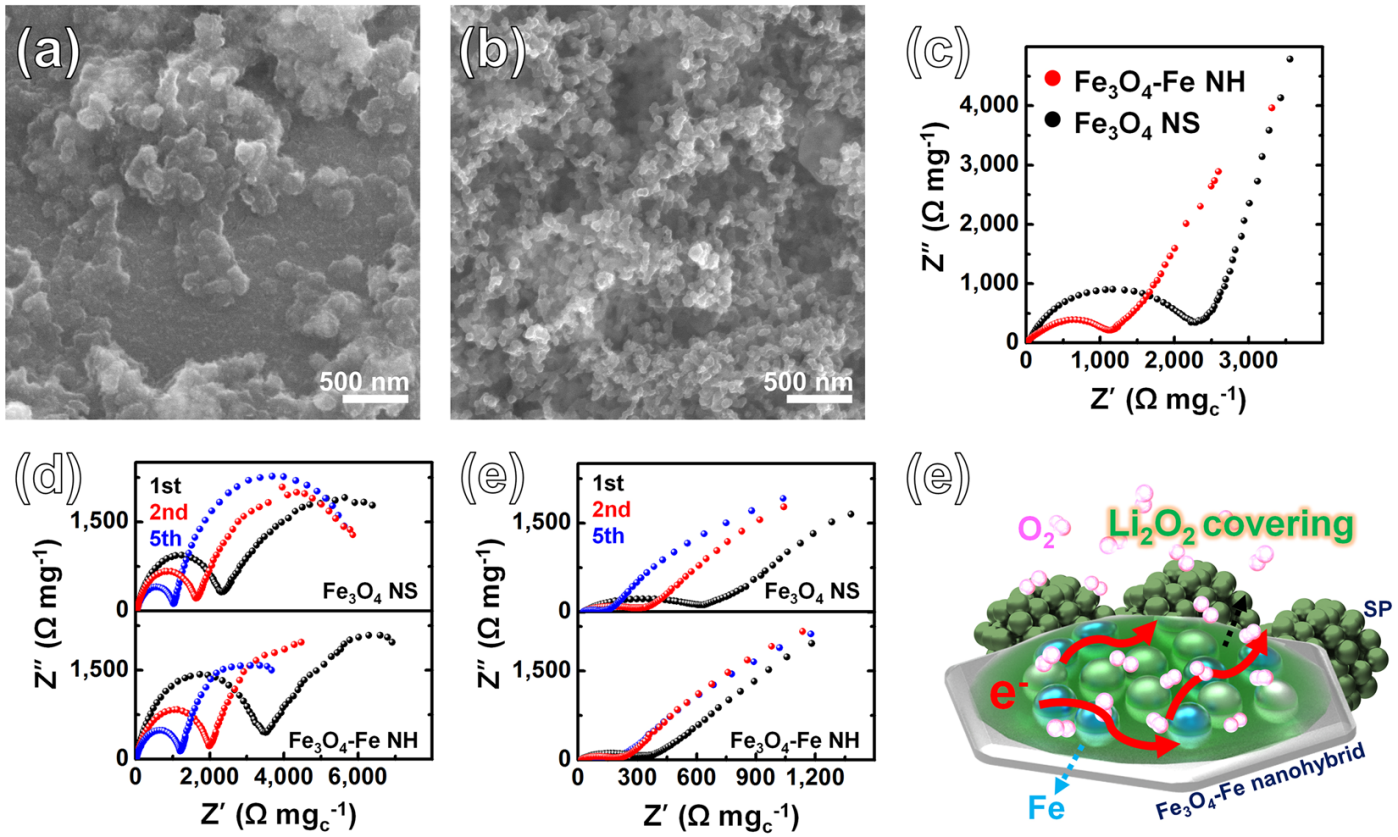
FESEM images of the Fe_3_O_4_-Fe NH electrode after (**a**) discharge and (**b**) charge process. Nyquist plots of the Fe_3_O_4_-Fe NH electrode and the Fe_3_O_4_ NS electrodes (**c**) at OCV, after (**d**) discharge process and (**e**) charge process. (**f**) Schematic representation emphasizing the electron transport paths through the metallic Fe.

To further investigate the enhanced electrocatalyst kinetics, we employed *ex-situ* FESEM after discharge–charge cycling and EIS analysis. The morphological changes after discharge and charge at a current rate of 500 mA g_c_^−1^ for the Fe_3_O_4_-Fe NH electrodes are shown in Fig. 5a and b. After cell discharge, large amounts of ORR aggregates (Li_2_O_2_) were formed on the surface of the electrode (Fig. 5a). During the subsequent charge process, the ORR aggregates disappeared at a charge capacity of 1,000 mA h g_c_^−1^ (Fig. 5b), indicating the high reversibility of the Fe_3_O_4_-Fe NH electrode.

The impedance behavior of both the Fe_3_O_4_-Fe NH and Fe_3_O_4_ NS electrodes in fresh cells is almost the same, as shown in Fig. 5c–e. After the discharge process, the resistances of both the Fe_3_O_4_-Fe NH and Fe_3_O_4_ NS electrodes increase significantly, which is due to the generation of insulating ORR products (Li_2_O_2_). Interestingly, after the charge process, the resistances of both electrodes are remarkably reduced (Fig. 5e), indicating that the formed insulating ORR products can be completely decomposed during the charge process, which is consistent with the SEM images of the Fe_3_O_4_-Fe NH electrode after the charge process (Fig. 5b).

A fitting model using equivalent circuits and the corresponding fitting values are depicted in Supplementary Table S3. In the Nyquist plots, the semicircle observed in the high-frequency region appears to be associated with film formation, mainly the accumulation of discharge products such as Li_2_O_2_ and the formation of a solid electrolyte interphase (SEI) layer owing to electrolyte decomposition on the electrodes. The intercept of this semicircle with the Z_real_ axis in the high-frequency region represents the total resistance of the electrolyte, separator, and electrical contacts (R_e_), whereas the diameter of the semicircle represents the interfacial resistance (R_i_), which is related to the coverage of Li_2_O_2_ and SEI layers. The semicircle in the medium-frequency region is associated with the time constant for charge-transfer resistance (R_ct_) at the electrode/electrolyte interface^36^. The Fe_3_O_4_ NS electrode has a significantly larger R_ct_ (2354.0 Ω mg_c_^−1^) than the Fe_3_O_4_-Fe NH electrode (1151.8 Ω mg_c_^−1^) at the open circuit voltage (OCV), suggesting that the highly conductive the Fe_3_O_4_-Fe nanohybrids decrease the electrode resistance. Similarly, the Fe_3_O_4_-Fe NH electrode has a significantly smaller R_ct_ than the Fe_3_O_4_ NS electrode after both the discharge and charge processes (see Supplementary Fig. S7). R_ct_ is inversely proportional to the rate coefficient of the chemical reaction, the porosity and the O_2_ concentration in the oxygen electrode. In Li–O_2_ battery, electrolytes should diffuse safely reduced oxygen species (O_2_^−^ or O_2_^2−^ by ORR: O_2_ + *n*e^−^ → O_2_^*n*−^, *n* = 1 or 2). As higher O_2_ concentration, *R*_CT_ is lower^37–39^. Decrease of R_CT_ indicates facilitated oxygen diffusion pathway and less agglomeration of the oxygen electrode. This means that the electrolyte and the electrode is activated. Therefore, the R_CT_ is remarkably reduced that the Fe_3_O_4_-Fe NH electrode is relatively stable cycling, and provide evidences for the superior performance of the Fe_3_O_4_-Fe NH electrode due to intimate contact and effective lithium ions and oxygen diffusion. Interestingly, R_i_ of the two electrodes show a similar tendency after discharge, and R_i_ of both electrodes disappear after the charging process of both electrodes due to sufficient OER activity, except for R_i_ after the first charge of the Fe_3_O_4_ NS electrode. This contributes to higher electronic conductivity, indicating the high ORR/OER activity of the Fe_3_O_4_-Fe NH electrode. We have evaluated the stability of Fe nanospheres through XPS. As shown in Supplementary Fig. S8, the Fe 2p XPS spectra of pristine Fe_3_O_4_-Fe NH electrode obviously indicate Fe^0^. The Fe 2p peaks at approximately 707 eV and 720 eV are associated, with the Fe^0^ 2p3/2 and 2p1/2 states of the Fe metal, respectively^40^. The Fe 2p peaks disappear after discharge process because it is covered by ORR products. After charge process, the Fe 2p peaks appear again. Therefore, it seems that even after discharge-charge cycling, relatively stable Fe remains rather than complete oxidation.

**Figure 6:**
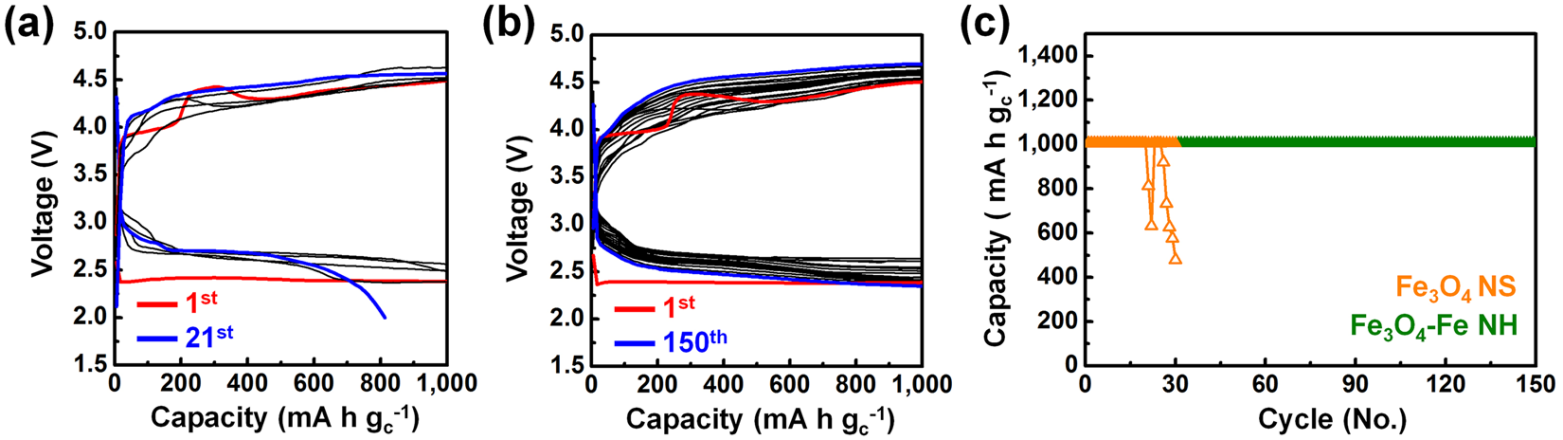
A high rate performances of the Fe_3_O_4_-Fe NH and the Fe_3_O_4_ NS electrode at a current rate of 2,000 mA g_c_^−1^. (**a**) The Fe_3_O_4_ NS electrode and (**b**) the Fe_3_O_4_-Fe NH electrode at a fixed capacity regime of 1,000 mA h g_c_^-1^ with a rate of 2,000 mA g_c_^−1^, (**c**) comparison of the discharge-charge specific capacity versus the cycle number of the Fe_3_O_4_-Fe NH electrode and the Fe_3_O_4_ NS electrodes.

Additionally, another considerable improvement of the Fe_3_O_4_-Fe NH electrode is a high rate performance in a Li–O_2_ battery, Fig. 6 show the galvanostatic discharge-charge cycling performance of the Fe_3_O_4_-Fe NH and Fe_3_O_4_ NS electrodes obtained in the fixed capacity regime of 1,000 mA h g_c_^−1^ at a rate of 2,000 mA g_c_^−1^. The Fe_3_O_4_-Fe NH electrode exhibited more stable cycling performance over 150 cycles compared to the Fe_3_O_4_ NS electrode. The Fe_3_O_4_ NS electrode exhibited a sudden deterioration of discharge capacity because its active sites were blocked by intrinsically electronically insulating ORR products, which could be confirmed again for electrode resistance problems (Fig. 5c–e). We note that the high catalytic activity of Fe_3_O_4_-Fe NH electrode, its result in an enhancement of the electronic conductivity, and it promote the decomposition of the insulating products.

## Conclusion

In this work, we fabricated the Fe_3_O_4_-Fe nanohybrid and the Fe_3_O_4_ nanosphere electrocatalysts for Li–O_2_ batteries via the electrical wire pulse process. The obtained the Fe_3_O_4_-Fe nanohybrids featured 0D spheres deposited on 2D flake composites, good dispersibility, and electronic conductivity. In Li−O_2_ battery tests at a current rate of 500 mA g_c_^−1^, the Fe_3_O_4_-Fe nanohybrids had a low overpotential of 1.39 V, a high capacity of 13,890 mA h g_c_^−1^, and stable cycling over 100 cycles at a fixed capacity of 1,000 mA h g_c_^−1^. Importantly, a comparison of the EIS results for the Fe_3_O_4_-Fe NH and Fe_3_O_4_ NS electrodes demonstrates the origin of the good ORR/OER activity.

## Method

### Materials and synthesis

Commercial Fe wire (0.2 mm in diameter) was purchased Nano Tech (Korea), and electrical pulse equipment (NTi-mini P, Nano Tech, Korea) was used to fabricate the Fe_3_O_4_-Fe nanohybrids and the Fe_3_O_4_ nanospheres. As a similar process reported in previous works^25, 26^. Fe-based aqueous nanocolloidal suspension could be successfully obtained. After completing the electrical wire pulse process with Fe wire, the obtained nanocolloidal suspension was allowed to settle for 3 days and divided into two classes of colloidal suspensions. Then, the selected nanocolloidal suspension was sonicated and filtered through a nylon membrane (Durapore, 0.22 mm, Millipore) several times, and subsequently dried at 120 °C for 8 hr.

### Characterization of Fe_3_O_4_-Fe nanohybrids and Fe_3_O_4_ nanospheres

The morphology and composition of the Fe_3_O_4_-Fe nanohybrids and the Fe_3_O_4_ nanospheres were investigated using transmission electron microscopy (TEM; Tecnai G2 F30 S-Twin, FEI), high angle annular dark field-scanning transmission electron microscopy with energy-dispersive X-ray spectroscopy (HAADF-STEM with EDS; JEM-2100F, JEOL, USA), field emission scanning electron microscopy (FESEM; S-4300, Hitachi), and X-ray photo-electron spectroscopy (XPS; PHI X-tool, ULVAC-PHI, Japan). The phase and crystal structure were characterized by X-ray diffraction (XRD; Ultima III, Rigaku). The particle size distribution was determined from dynamic light scattering (DLS) analyses using a particle size analyzer (PSA; ELSZ-1000, Otsuka Electronics Korea Co. Ltd.).

### Electrochemical performance of Li−O_2_ cells

The electrochemical performance of the Fe_3_O_4_ nanospheres and the Fe_3_O_4_-Fe nanohybrids was evaluated using Swagelok-type cells. The electrode was prepared by mixing each Fe nanopowder (45%) with Super P carbon black (45%) and carboxymethyl cellulose (10%; CMC, Aldrich, Average Mw ~700,000). The obtained slurry was spread onto nickel foam, and the loading weight of the electrode was adjusted to above 0.2 mg of super P carbon black per cm^2^ The Li–O_2_ cells were assembled in an Ar-filled glove box. The cells consisted of a lithium foil as the anode, a glass microfiber filter (Celgard 2400, Wellcos) as the separator, 1 M LiNO_3_ (Alfa Aesar, anhydrous, ≥99.999%) in N,N-dimethylacetamide (DMAc; Alfa Aesar, anhydrous, ≥99.8%) as the electrolyte, the electrode, and carbon cloth (W0S1002, CeTech) as a gas diffusion layer. All measurements were conducted in 1.5 atm dry oxygen to avoid any negative effects of humidity and CO_2_. The assembled cells were tested with an automatic battery cycler (WBCS 3000, WonAtech) in a voltage window of 2.0–4.8 V. Electrochemical impedance spectroscopy (EIS) was performed with an electrochemical workstation (Ivium-n-Stat electrochemical analyzer, Ivium Technologies B. V.). The impedance response was collected by applying AC voltages of 10 mV while maintaining a constant DC voltage in the frequency range of 0.01 Hz to 100 kHz. All the above measurements were conducted at room temperature.

## Electronic supplementary material

Supplementary Info
