# Supplementary material for: Fe-based hybrid electrocatalysts for nonaqueous lithium-oxygen batteries
Source: Sci Rep. 2017 Aug 25;7:9495. doi: 10.1038/s41598-017-09982-9 (PMC5573321; doi:10.1038/s41598-017-09982-9)
Supplement: Supplementary file 1 — Supplementary Info [file 41598_2017_9982_MOESM1_ESM.pdf]

Supplementary Information

Fe-based hybrid electrocatalysts for nonaqueous lithium-oxygen batteries

Seun Lee[1,3], Gwang-Hee Lee[1,3], Hack Jun Lee[1], Mushtaq Ahmad Dar[2], & Dong-Wan Kim[1,*]

[1] School of Civil, Environmental and Architectural Engineering, Korea University, Seoul 02841, Republic of Korea

[2] Center of Excellence for Research in Engineering Materials, Advanced Manufacturing Institute, College of Engineering, King Saud University, Riyadh 11421, Saudi Arabia

[3] These authors contributed equally to this work.

* Correspondence and requests for materials should be addressed to D.W.K. (E-mail: dwkim1@korea.ac.kr)

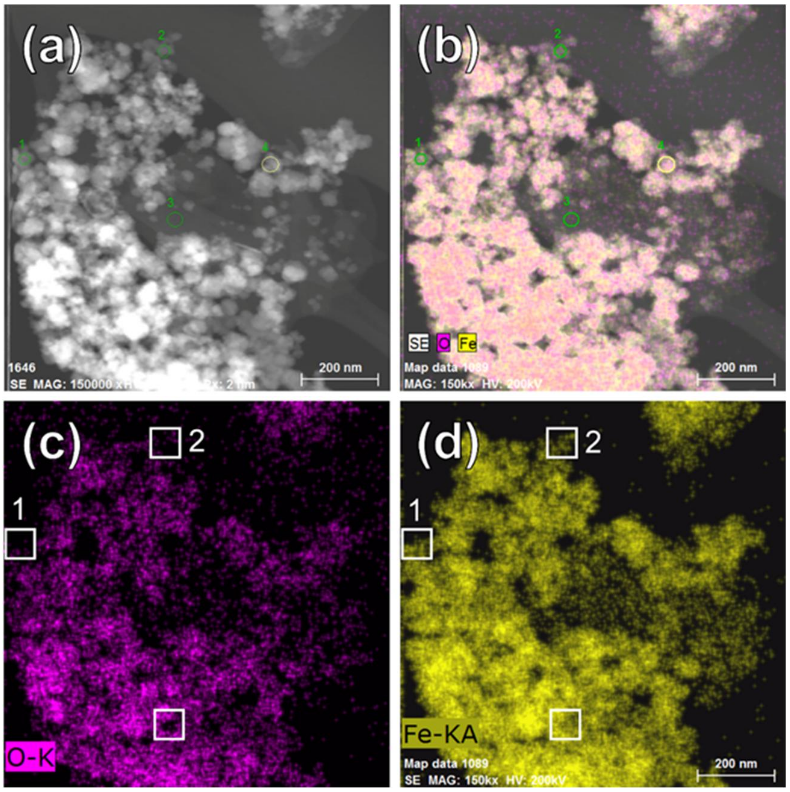

Figure S1. (a) HAADF-STEM images and (b–d) EDS element mapping analysis of the Fe_3O_4-Fe nanohybrids.

Table S1. EDS results for the Fe_3O_4-Fe nanohybrids of Figure S1.

Position	Atomic %	
	O	Fe
**1**	0.00	100.00
**2**	0.00	100.00
**3**	59.95	40.05
**4**	60.17	39.83

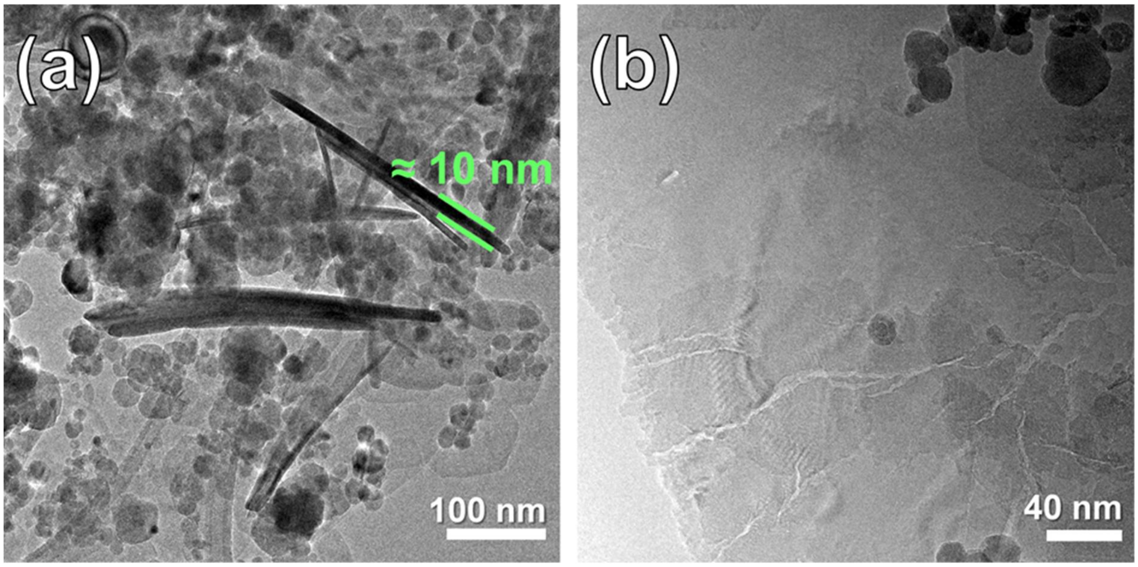

Figure S2. (a) Low-magnitude and (b) high-magnitude TEM image of the Fe_3O_4-Fe nanohybrids.

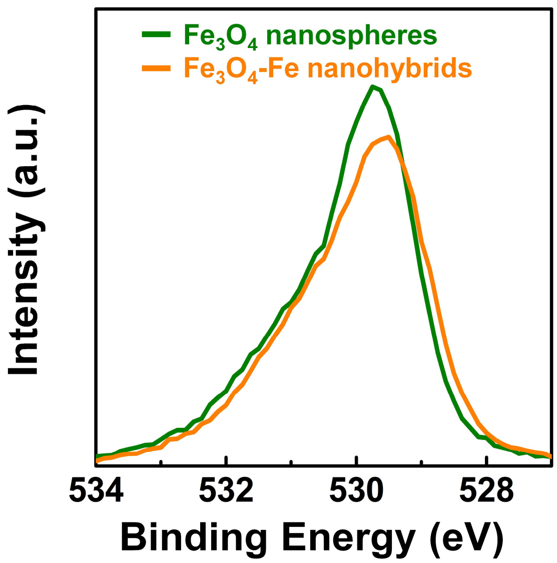

Figure S3. O 1s XPS spetra of the Fe_3O_4-Fe nanohybrids and the Fe_3O_4 nanospheres.

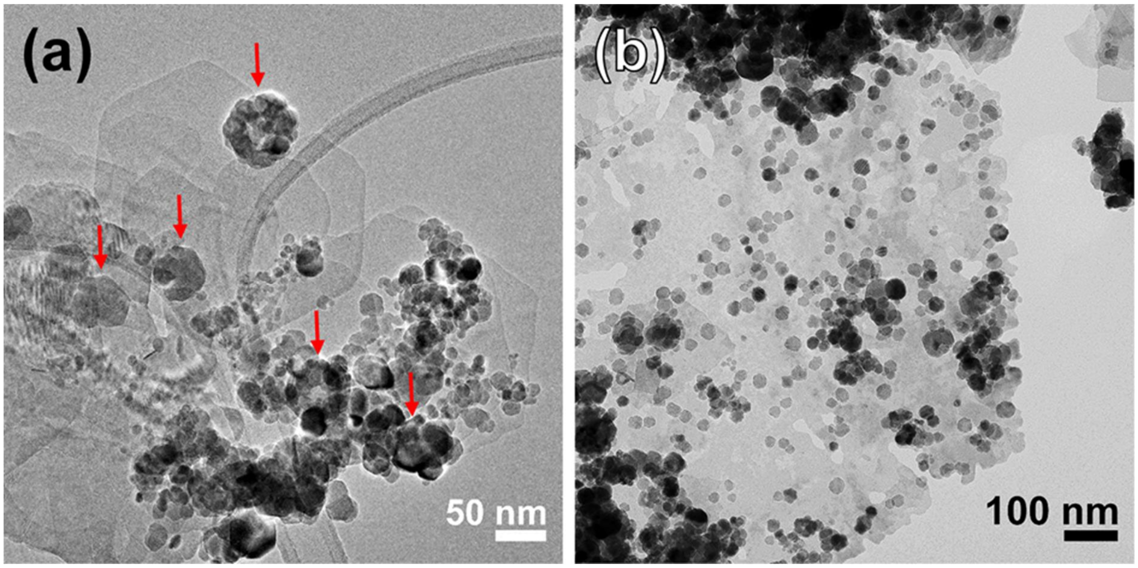

Figure S4. TEM images of the detailed growth stage. Oriented attachment growth to form a polycrystalline structure and subsequent recrystallization were shown.

Table S2. Average particle size of the Fe_3O_4-Fe nanohybrids and the Fe_3O_4 nanospheres and theoretical density of Fe_3O_4 and Fe.

	Fe_3O_4-Fe nanohybrids	Fe_3O_4 nanospheres	Element	
			Fe_3O_4	Fe
Average (nm)	229.5	3,753.2	-	-
Theoretical denstiy (g cm^{-3})	-	-	5.235	7.874

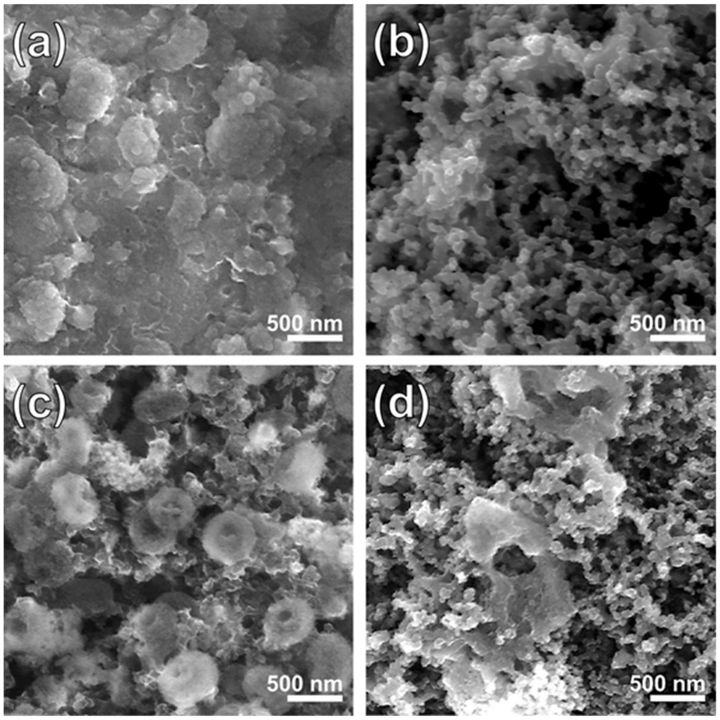

Figure S5. FESEM images of the Fe_3O_4-Fe NH electrode after (a) discharge and (b) charge process. FESEM images of the Fe_3O_4 NS electrode after (c) discharge and (d) charge process.

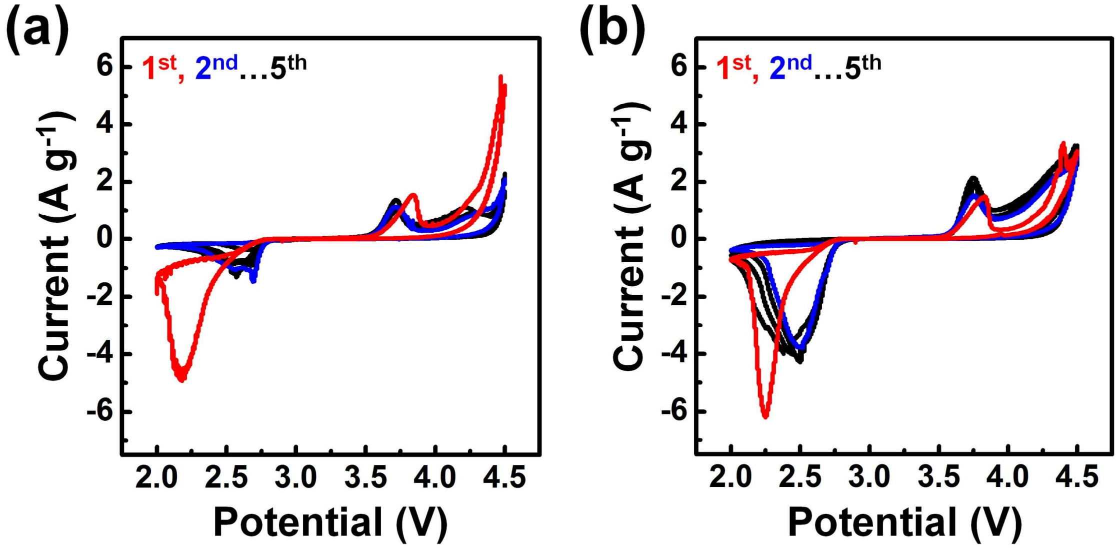

Figure S6. CV measurements of (a) the Fe_3O_4 NS electrode and (b) the Fe_3O_4-Fe NH electrode at a scanning rate of 0.05 mV s^{-1} in a voltage window of 2.0–4.5 V.

Table S3. RC equivalent circuit model and corresponding fitting values of the Fe_3O_4-Fe NH and Fe_3O_4 NS electrodes.

Electrode	Cycle		RC equivalent circuit model	R_e ($\Omega\ mg^{-1}$)	R_i ($\Omega\ mg^{-1}$)	R_{ct} ($\Omega\ mg^{-1}$)
Fe_3O_4 NS	OCV		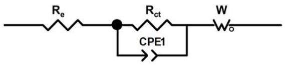	32.9	-	2354.0
	Discharge	1st	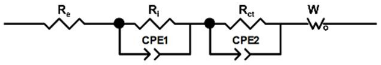	31.2	2321.4	6164.7
		2nd		27.4	1667.9	5135.5
		5th		27.1	1042.2	5296.7
	charge	1st	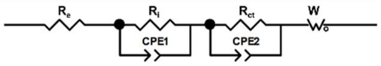	28.1	607.9	3934.8
		2nd	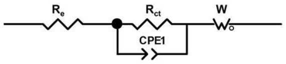	24.0	-	302.2
		5th		20.9	-	121.4
Fe_3O_4-Fe NH	OCV		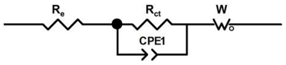	1.6	-	1151.8
	Discharge	1st	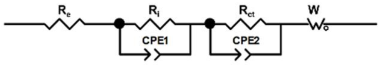	16.8	3462.1	5258.2
		2nd		13.3	2051.2	4690.9
		5th		13.3	1241.4	3771.1
	charge	1st	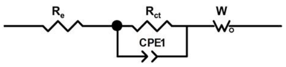	11.3	-	333.7
		2nd		12.1	-	170.2
		5th		12.1	-	167.1

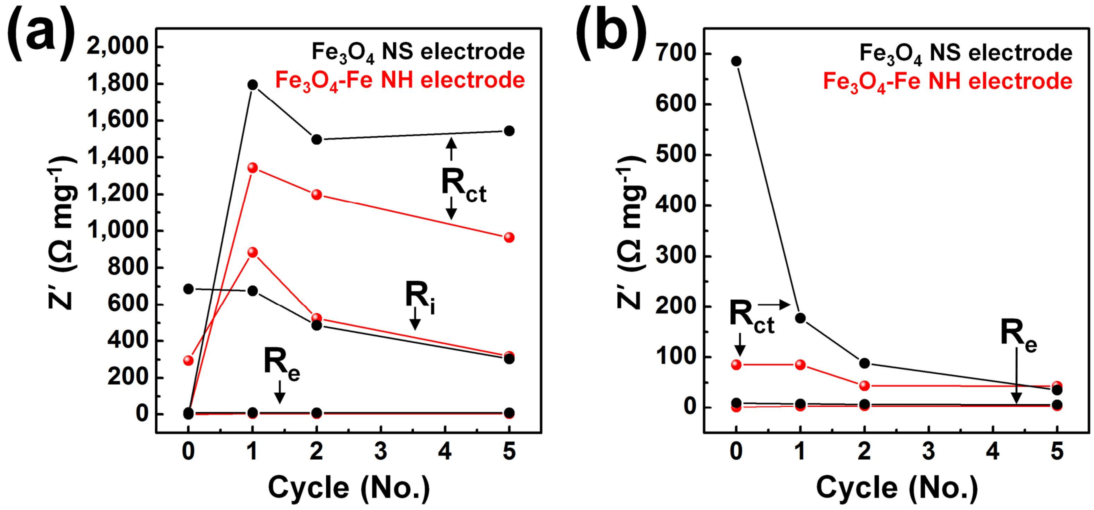

Figure S7. Fitting resistance values of the Fe_3O_4-Fe NH and Fe_3O_4 NS electrodes after (a) discharge (including OCV) and (b) charge processes.

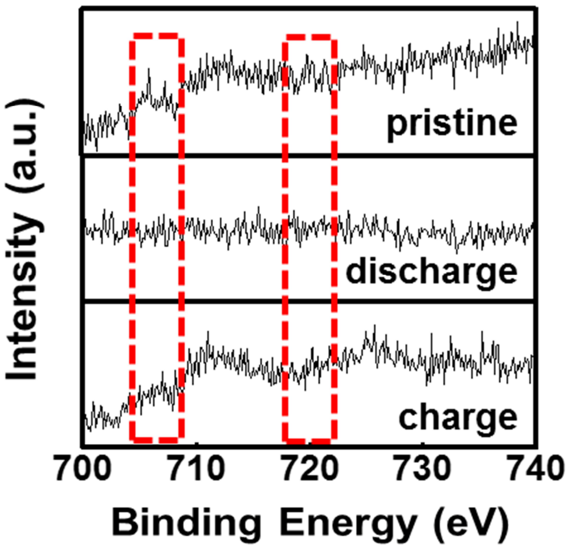

Figure S8. Fe 2p XPS spectra of the Fe_3O_4-Fe NH electrode after cycle tests at the open circuit voltage.
